# Response to Nguyen et al.'s letter regarding “Anteriolateral versus anterior–posterior electrodes in external cardioversion of atrial fibrillation: A systematic review and meta‐analysis of clinical trials”

**DOI:** 10.1002/clc.24253

**Published:** 2024-03-14

**Authors:** Karam R. Motawea, Abdulqadir J. Nashwan

**Affiliations:** ^1^ Faculty of Medicine Alexandria University Alexandria Egypt; ^2^ Department of Nursing Hamad Medical Corporation Doha Qatar

Dear Editor,

We would like to thank Nguyen et al. for engaging with our systematic review and meta‐analysis^1^ and for your critical observations.^2^ Regarding the omission of four studies, our inclusion criteria were rigorously followed, which led to the exclusion of studies that did not meet these predefined standards. Specifically, three of these studies were either not in English or inaccessible in full text, preventing their inclusion.^3^, ^4^ The fourth study focused primarily on a subgroup analysis of patients with obesity, which did not align with our broader inclusion criteria encompassing all patient demographics, not limited to specific conditions like obesity.^5^

Concerning the inclusion of a prospective study, it appears there was a misunderstanding about the nature of the study we included. The study in question was a prospective interventional study, effectively functioning as a non‐randomized clinical trial, which falls within our criteria of including both randomized and non‐randomized clinical trials.^6^ This inclusion aligns with our commitment to a comprehensive analysis of clinical trials relevant to our research question.

As for the issue of data extraction, particularly regarding the studies by Alp et al.^7^ and Botto et al.,^8^ our methodology adhered strictly to the principles of accurate data appraisal. The overall cardioversion rates utilized in our analysis were directly reflective of the outcomes post‐application of DC shock and high energy, consistent with the intentions of the original studies. We believe this approach maintains the integrity of our analysis and supports the validity of our findings.

We acknowledge the value of constructive critique and the importance of rigorous debate in advancing scientific understanding. As the field moves forward, especially with ongoing trials like NCT05511389, we anticipate further clarification on optimal practices, including electrode pad placement. Our study contributes to this ongoing dialogue, and we advocate for continued research that incorporates best practices alongside new interventions.

## CONFLICT OF INTEREST STATEMENT

The authors declare no conflict of interest.
